# Early identification of autism spectrum disorder in preschoolers by static and dynamic amplitude of low-frequency fluctuations features

**DOI:** 10.3389/fnhum.2025.1513200

**Published:** 2025-04-10

**Authors:** Kanghui Yu, Shoujun Xu, Shishun Fu, Kelei Hua, Yi Yin, Qiang Lei, Jinwu Liu, Yunfan Wu, Guihua Jiang

**Affiliations:** ^1^The Second School of Clinical Medicine, Southern Medical University, Guangzhou, China; ^2^Department of Medical Imaging, The Affiliated Guangdong Second Provincial General Hospital of Jinan University, Guangzhou, China; ^3^Department of Radiology, Shenzhen Children’s Hospital, Shenzhen, China; ^4^Department of Medical Imaging, Central Hospital of Wuhan, Wuhan, China

**Keywords:** autism spectrum disorder, preschooler, resting-state fMRI, static, dynamic, amplitude of low-frequency fluctuation

## Abstract

**Objectives:**

Early identification and timely intervention is critical for young children with autism spectrum disorder (ASD). The current study aims to explore potential disparities in static and dynamic intrinsic brain function in preschoolers with ASD, and uncover underlying neural underpinnings that can be used for facilitating the identification of ASD.

**Materials and methods:**

Static and dynamic amplitude of low-frequency fluctuations (ALFF) of 73 ASD preschoolers and 43 age-matched typically developing individuals (TDs) were extracted and compared to identify differences in intrinsic brain local connectivity associated with ASD. The dynamic ALFF (dALFF) utilized a sliding window technique that integrates static ALFF (sALFF) to gauge the variance of local brain activity over time. A receiver operating characteristic (ROC) analysis was conducted to evaluate the potential diagnostic capability of the sALFF and dALFF metrics in identifying ASD.

**Results:**

Compared with TDs, ASD preschoolers exhibited lower levels of sALFF in the left middle temporal gyrus, medial orbitofrontal cortex, precuneus and reduced dALFF values in the left inferior orbitofrontal cortex, middle temporal gyrus. ROC analysis indicated that sALFF and dALFF could distinguish preschoolers with ASD from TDs with the areas under the curve (AUC) of 0.848 and 0.744 (*p* < 0.001), and their combination showed an increased accuracy with the AUC of 0.866 (*p* < 0.001). Nevertheless, there were no linear correlation between the ALFF values in children with ASD and clinical scales.

**Conclusion:**

The findings suggest an association of regional left brain dysfunction with ASD in preschoolers. The values of sALFF and dALFF, particularly in the middle temporal gyrus, could act as possible indicators for the early detection of ASD.

## Introduction

Autism spectrum disorder (ASD) is a complex and heterogeneous neurodevelopmental disease that originates in early childhood. It is characterized by impaired social communication, repetitive stereotyped behaviors and narrowed interests (American Psychiatric Association, 2013). The rising prevalence of ASD (Hirota and King, 2023; Sun et al., 2019) poses a global public health issue and economic burden to patients and their families (Xu S. et al., 2019). Global Burden of Disease (GBD) statistics indicate that there are over 603,750 children under the age of 5 with ASD worldwide (Yi et al., 2024). Therefore, early diagnosis and timely intervention for ASD, is of great significance, especially for preschoolers with malleable brain development.

Although pathological mechanisms of ASD remain elusive, it is widely accepted that insufficient neuronal connections and disruption of spontaneous neural activity contribute to ASD development (Saxena et al., 2020). Moreover, ASD is distinguished by atypical trajectories of brain maturation (Guo et al., 2017), which are linked to varied neural development of brain anatomy, function, and connectivity (Ecker et al., 2015). As an effective non-invasive brain imaging technique, resting-state functional magnetic resonance imaging (rs-fMRI) can capture spontaneous low-frequency functional signals through blood oxygen level-dependent (BOLD) responses, primarily reflecting brain activities through intrinsic functional connections. Previous rs-fMRI studies on ASD predominantly focused on whole-brain functional connectivity (FC) analysis, with varied findings on regional consistency and FC changes in ASD (Di Martino et al., 2013; Wiśniowiecka-Kowalnik and Nowakowska, 2019). Unlike FC, which evaluates the synchronized BOLD time series across specific brain regions or networks, the amplitude of low-frequency fluctuations (ALFF) quantifies the intensity of the BOLD signal in each voxel. It is capable of mirroring the degree of neural activity that occurs spontaneously in particular brain areas, possessing temporal constancy and test–retest dependability (Küblböck et al., 2014; Zuo and Xing, 2014). Supekar et al. (2013) revealed that, compared to TDs, children with ASD showed increases of the mean global FC, accompanied by increased mean ALFF. Li et al. (2021) found ALFF values in the cerebellar region of adolescent ASD patients are significantly correlated with some clinical features. Yue et al. (2022) conducted an ALFF and regional homogeneity (ReHo) in prepubertal boys. Compared to the TD group, the ASD group exhibited a reduced ALFF value in the left inferior parietal lobule, along with decreased ReHo values in the left lingual gyrus, left superior temporal gyrus, left occipital gyrus, and right cuneus. Another study (Karavallil Achuthan et al., 2022) utilized a combination of ALFF and fractional amplitude of low-frequency fluctuation (fALFF) as a measurement method to compare intrinsic brain activity between children with and without autism. Focusing on seven large-scale brain networks that play a pivotal role in both resting and task-related brain functions, they discovered that ALFF, which partially involves social and affective cognitive regions, increased in autism patients, while it decreased in regions including key nodes of the default mode network (DMN). Most studies tend to focus on a single age group or neglect developmental effects. Guo et al. (2017) investigated the atypical developmental trajectory of spontaneous brain activity in individuals with autism spectrum disorder across a broad age range. Their findings revealed that the ALFF values in the precuneus and left occipital gyrus were significantly reduced in the ASD group across all developmental stages. Furthermore, the abnormal ALFF values were directly correlated with social deficits in ASD, indicating that abnormal spontaneous brain activity might be a potential mechanism underlying social deficits in ASD.

Since the resting brain works as a dynamic system, exhibiting a non-static spatiotemporal functional organization (James et al., 2019; Li J. et al., 2018), researchers can obtain a landscape of fluctuation pattern of brain activity amplitudes as time progresses. Consequently, some scholars (Fu et al., 2018) introduced dynamic ALFF (dALFF) that integrates static ALFF (sALFF) with the sliding window approach to assess the variation of ALFF across different time intervals. It has been broadly utilized to investigate pathological alterations in brain activity of patients suffering from diverse neuropsychiatric disorders (Karavallil Achuthan et al., 2022; Liang et al., 2023; Yang et al., 2022). Prior investigations have demonstrated that the dynamic analysis of brain function serves as a crucial approach for uncovering the origin and development mechanisms of brain disorders (Cui et al., 2019; Song et al., 2023; Zheng et al., 2021; Zhu et al., 2024). Previous research primarily concentrated on the static brain function of ASD, overlooking the dynamic characteristics of spontaneous brain activity in the temporal dimension. Recently, there have been sporadical studies examining the dynamic changes in local brain activity among ASD patients. For instance, Yue et al. (2023) combined with dALFF and dynamic regional homogeneity (dReHo) methods, to investigate the dynamic characteristics of regional neurological function in adult ASD patients. The findings revealed a significant increase in dALFF variability in the left middle occipital gyrus, left superior parietal gyrus, left precuneus, left inferior temporal gyrus, and right inferior frontal gyrus, orbital part among adult ASD patients. Another study (Wu et al., 2023) also utilized dReHo and dALFF methods to explore the disparities in dynamic brain activity between toddlers aged 1–3 years with ASD and language development delay (LDD), and found that the dALFF values in the right MTG and right precuneus were notably lower in the ASD group compared to the LDD group, indicating smaller low-frequency fluctuation amplitudes in these regions among ASD children, potentially linked to their language processing deficits. However, the aforementioned researches are confined to a single dynamic change and fail to simultaneously analyze both dynamic and static indicators to compare their similarities and differences.

On the other hand, multiple studies have suggested that age has a significant impact on ALFF values (Guo et al., 2017; Mei et al., 2022). Recent investigations into the exploration of dynamic brain activities in ASD mainly focused on toddlers or adult patients, with varying results (Wu et al., 2023; Yue et al., 2023), while ALFF features in preschoolers remain to be characterized. As far as we know, this study is the first to apply sALFF and dALFF in a comparative study between preschool children with ASD and those without. We aim to identify ASD-specific ALFF features that could be used as potential diagnostic biomarkers for ASD. These findings will offer novel insights into neuroimaging processes related to social and language difficulties, restricted interests and repetitive behaviors in preschoolers with ASD, and facilitate early identification and intervention of ASD.

## Materials and methods

### Participants

This study recruited 86 children with ASD and 54 age- and sex-matched typically developing individuals (TDs). The study was approved by the Ethics Committee at the Second Clinical Medical College of Southern Medical University. It was in line with the principles stated in the Helsinki Declaration and its subsequent revisions or comparable ethical norms. Guardians of every participant provided consent after having been comprehensively informed about the aim of the study. The inclusion criteria were clearly defined: (1) Han Chinese ethnicity; (2) aged 2–6 years; (3) right-handed boys or girls; (4) no prior history of head trauma, mental and neurological ailments (including epilepsy and Tourette syndrome), psychiatric disorders (for example, childhood disintegrative disorder, obsessive-compulsive disorder, Asperger’s syndrome, or selective mutism), or genetic defects (such as Rett syndrome and Fragile X syndrome). Exclusion criteria included chronic systemic diseases, contraindications for MRI scanning, or use of antipsychotic medications for all participants. The final ASD group comprised 73 cases (a total of 13 cases were excluded, with 12 cases younger than 2 years old and 1 case older than 6 years old). Meanwhile, the control group consisted of 43 individuals (a total of 11 cases were excluded, including 10 cases younger than 2 years old and 1 case older than 6 years old).

### Clinical assessment

Through clinical interviews, all patients satisfied the Diagnostic and Statistical Manual of Mental Disorders, 5th Edition (DSM-V) criteria for ASD and completed both the Autism Behavior Checklist (ABC) (Krug et al., 1980) and the Childhood Autism Rating Scale (CARS) (Schopler et al., 1980), which are the primary tools for diagnosing and screening ASD in China. The ABC is filled out by the parents of the subjects and is appropriate for individuals ranging from 8 months to 28 years, whereas the CARS is evaluated by qualified psychologists and is intended for individuals who are 2 years old and above. The Developmental Quotient (DQ) is a commonly used indicator to test the neurodevelopment of preschoolers, which reflects the social and psychological abilities of children. The DQ is assessed using the Developmental Diagnosis Scale for 0- to 6-year-old children, with a score of less than 70 indicating low development.

### Image acquisition

Image acquisition is performed by experienced radiographers. Prior to the scan, all subjects are orally administered or given an enema of 0.5% chloral hydrate at a dose of 0.5 mL/kg (maximum dose of 10 mL) to induce and maintain sedation. Throughout the scanning process, each child requires a caregiver or guardian present.

MRI examinations are performed with a Siemens Skyra 3.0 T MRI scanner, which has an eight-channel head coil, in the Department of Radiology at Shenzhen Children’s Hospital. To reduce head movement and operator noise, each participant is placed in a supine position, supported by foam padding and fitted with headphones. The following are the rs-fMRI acquisition parameters: echo time (TE)/repetition time (TR), 30 ms/2 s; slice thickness of 3.6 mm with a 0.72 mm gap; field of view (FOV), 230 mm × 230 mm; flip angle, 90°; matrix, 64 × 64; number of slices, 35. A total of 240 volumes are acquired within 8 min. Meanwhile, the high-resolution T1-weighted structural MRI images had the following parameters: 176 sagittal sections; TR = 2,300 ms, TE = 2.25 ms, TI = 1900 ms; Flip angle, 8°; acquisition matrix: 256 × 256, FOV: 256 × 256 mm, and layer thickness was 1 mm. Once the MRI scan is completed, the images of each participant are examined to ensure that they fulfill the study’s criteria.

### Data preprocessing

The DPABI software^1^ (Yan et al., 2016) and the SPM12 software package, which run on the MATLAB R2016a platform (MathWorks Inc., Natick, MA, USA), were utilized for preprocessing the rs-fMRI data. The primary stages involved in the preprocessing of rs-fMRI data included: (1) eliminating the initial 10 time points to ensure the acquisition of stable scans; (2) slice-time correction; (3) motion correction: participants with a maximum displacement greater than 2 mm and angular motion greater than 2° throughout the fMRI scan were excluded to minimize the impact of head motion; the average framewise displacement (FD) was taken as the head motion parameter, and participants whose FD exceeded 0.5 were excluded (Jenkinson et al., 2002); (4) normalization of all functional image data related to subjects to the Montreal Neurological Institute (MNI) space, along with resampling the images to a voxel size of 3 mm × 3 mm × 3 mm (Fonov et al., 2011); (5) elimination of linear trends; (6) regression of 24 head motion parameters, global signals, white matter and cerebrospinal fluid signals; (7) application of band-pass filtering (0.01–0.08 Hz). To enhance the signal-to-noise ratio, a Gaussian filter with a full width at half maximum of 6 mm was employed to smooth the fMRI data. Finally, both sALFF and dALFF were computed at the voxel level.

### Static and dynamic ALFF computation

ALFF mainly functions to depict the resting-state functional activity within each brain voxel. Following standardization, the time series data of fMRI is transformed into the frequency domain using a Fast Fourier transform. Then, sALFF values are calculated using the mean square root of the power spectrum at frequencies ranging from 0.01 to 0.08 Hz (Yu-Feng et al., 2007). The dALFF is determined through the sliding window approach using the DPABI software. Window length is critical for calculating resting-state dynamics. In this study, by using a window length of 50 TRs (100 s) and a step size of 5 TRs (10 s), optimal parameters were established, which achieved a balance between seizing reliable dynamics and ensuring stable correlations among the regions involved in the dALFF calculation (Cui et al., 2019; Ma et al., 2020; Song et al., 2023). In each window, ALFF maps are calculated, and the variance among these maps throughout all windows serves as an indicator of dynamics. To quantitatively assess and contrast the dynamic features of dALFF over time, we measured the variance of the ALFF maps using standard deviation (SD) to evaluate the temporal variability of the brain activity amplitude. Subsequently, both the sALFF and dALFF maps are standardized to z-scores and subjected to smoothing using a Gaussian kernel with a full-width at half-maximum (FWHM) of 6 mm to facilitate further statistical evaluation.

### Statistical analysis and correlation analysis

The clinical data were analyzed statistically with IBM SPSS Statistics 25, and differences were regarded as significant when *p* < 0.05. With age, DQ and FD as covariates, a two-sample t-test was applied to compare the estimation values of sALFF and dALFF were compared between children with ASD and TDs. After the Gaussian Random Field (GRF) correction was applied, with a voxel-level *p* < 0.001 (two-tailed) and a cluster-level *p* < 0.05 (two-tailed), statistical significance was achieved. Additionally, Pearson correlation analysis was conducted to examine the associations between sALFF or dALFF values and CARS, ABC.

In addition, by using binary logistic regression, the predictive probabilities of sALFF and dALFF values in abnormal brain regions as well as their combination for diagnosing ASD were determined. In the step of the logistic regression step, we incorporated age, DQ and FD as covariates to eliminate the potential influence factors, Receiver operating characteristic (ROC) curves were adopted to evaluate the predictive accuracy of sALFF dALFF and their combination, which was quantified by the area under the curve (AUC). The mean sALFF and dALFF values for all voxels that differed significantly were extracted separately. ROC curves were constructed using abnormal sALFF and dALFF as features. An AUC that exceeds 0.9 signifies outstanding diagnostic effectiveness, while a range between 0.7 and 0.9 reflects good diagnostic capability. An AUC falling between 0.5 and 0.7 denotes inadequate diagnostic performance, and any value at or below 0.5 suggests no diagnostic utility (Niu et al., 2023).

## Results

### Demographics of participants

Table 1 details the participants’ demographics and clinical scale scores of the ASD and TD groups. No notable discrepancies in gender (*p* = 0.521) age (*p* = 0.712) or head motion (*p* = 0.065) were found between the two groups. The DQ value was lower in the ASD group than the TD group (*p* < 0.001), which aligns with the diagnosis for ASD. The values for CARS and ABC are not available in the TD group.

**Table 1:** Demographic and clinical characteristics of participants.

Variables	ASD (*n* = 73)	TD (*n* = 43)	*p*-value
Gender (B/G)	57/16	33/10	0.521^*^
Age (years)	3.32 ± 0.97	3.39 ± 0.81	0.712^#^
Mean FD	0.051 ± 0.028	0.041 ± 0.021	0.065^#^
DQ	56.44 ± 5.87	86.37 ± 5.87	0.000^#^
ABC	55.64 ± 9.48		
CARS	35.42 ± 2.99		

Values are presented as mean ± standard deviation. ASD, Autism spectrum disorder; HC, healthy control; B, boy; G, girl; FD, framewise displacement; DQ, Developmental Quotient; ABC, Autism Behavior Checklist; CARS, Childhood Autism Rating Scale.

* The *p*-value was obtained with a chi-squared test.

# The *p*-value was obtained with a two-sample t-test.

### Difference of sALFF and dALFF between ASD and TD groups

**Figure 1:**
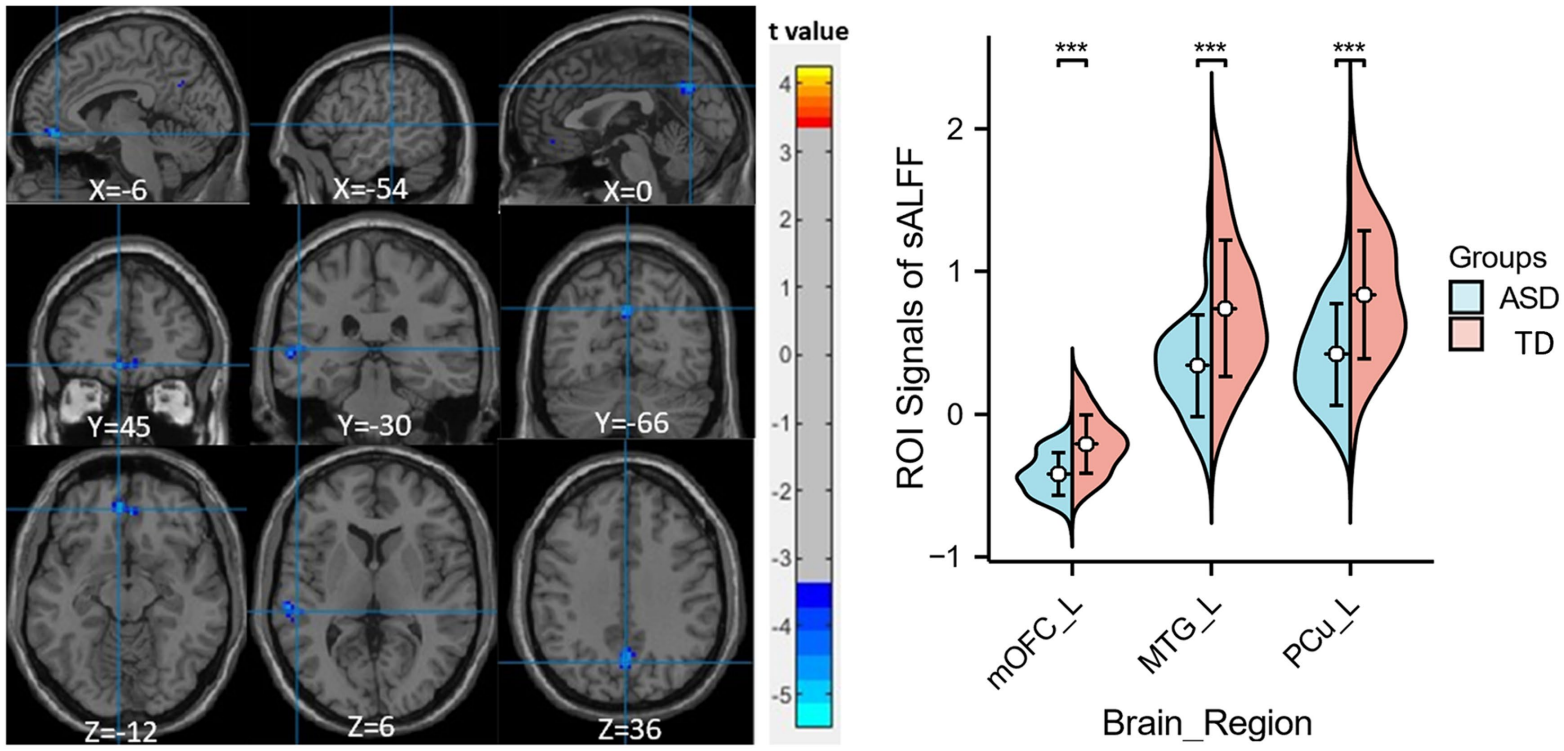
Significant sALFF differences between ASD children and TDs. A two-sample t-test was conducted to evaluate the sALFF values in children with ASD compared to TDs. Markedly decreased sALFF in left medial orbitofrontal cortex, left middle temporal gyrus and left precuneus were found in children with ASD (blue colors). X, Y, and Z stand for the coordinates of main peak positions of the abnormal brain region in the Montreal Institute (MNI) space. Distributions of sALFF [if Figure 1 legend; or dALFF, if Figure 2 legend] values for ASD children and TDs are shown on the right for all significant clusters, ***p < 0.001. L, left; mOFC, medial orbitofrontal cortex; MTG, middle temporal gyrus; PCu, precuneus.

**Figure 2:**
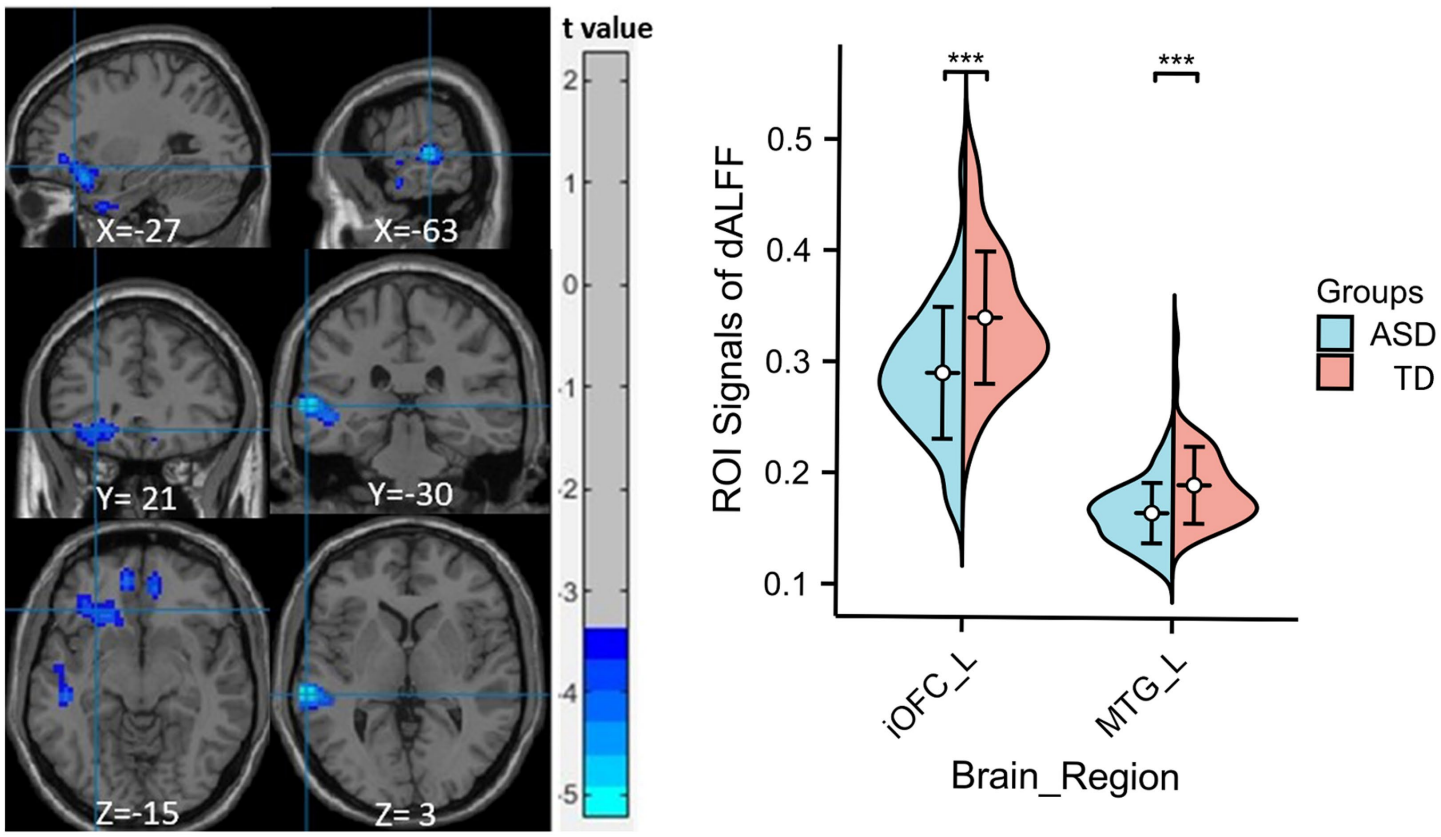
Significant sALFF differences between ASD children and TDs. A two-sample t-test was conducted to evaluate dALFF values in children with ASD compared to TDs. Markedly decreased dALFF in left inferior orbitofrontal cortex and left middle temporal gyrus were found in children with ASD (blue colors). X, Y, and Z stand for the coordinates of main peak positions of the abnormal brain region in the Montreal Institute (MNI) space. The ROI signal values for altered regional brain regions between ASD children and TDs is shown on the right, ***p < 0.001. L, left; iOFC, inferior orbitofrontal cortex; MTG, middle temporal gyrus.

Compared to the TD group, children with ASD exhibited a marked reduction in sALFF within the left medial orbitofrontal cortex (mOFC), the left middle temporal gyrus (MTG), and the left precuneus (PCu) (Table 2 and Figure 1), while decreased dALFF was detected in the left inferior orbitofrontal cortex (iOFC) and the left MTG (Table 2 and Figure 2).

**Table 2:** Differences of sALFF and dALFF between ASD and TD groups.

Indices	Brain regions	Cluster size	Peak MNI coordinates			AAL	T-values
			X	Y	Z		
Static	L mOFC	18	−6	45	−12	Frontal_Med_Orb_L	−5.5049
ALFF	L MTG	17	−54	−30	6	Temporal_Mid _L	−4.9527
	L PCu	26	0	−66	36	Precuneus _L	−4.5912
Dynamic	L iOFC	140	−27	21	−15	Frontal_Inf_Orb_L	−4.5047
ALFF	L MTG	228	−63	−30	3	Temporal_Mid_L	−5.2261

L, left; ALFF, amplitude of low frequency fluctuations; MNI, Montreal Neurological Institute; AAL, anatomical automatic labeling; mOFC, medial orbitofrontal cortex; MTG, middle temporal gyrus; PCu, precuneus; iOFC, inferior orbitofrontal cortex.

### Correlation between ALFF and clinical scales and ROC curve analysis of ALFF values

**Figure 3:**
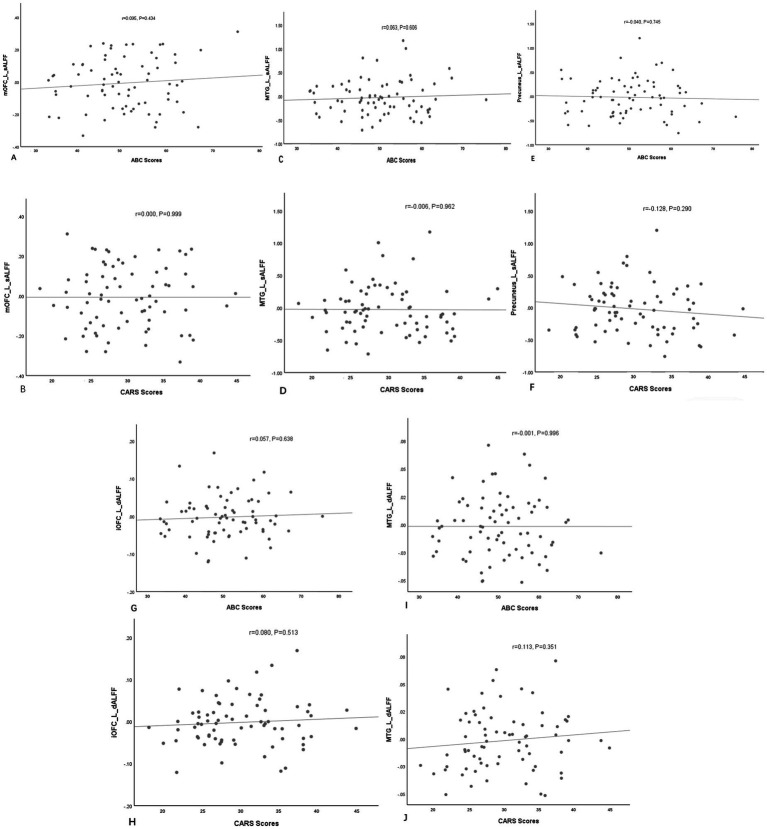
The correlations between sALFF **(A–F)** or dALFF **(G–J)** values and the ABC/CARS scores in the ASD group. No notable correlation was found. L, left; mOFC, medial orbitofrontal cortex; MTG, middle temporal gyrus; PCu, precuneus; iOFC, inferior orbitofrontal cortex.

**Figure 4:**
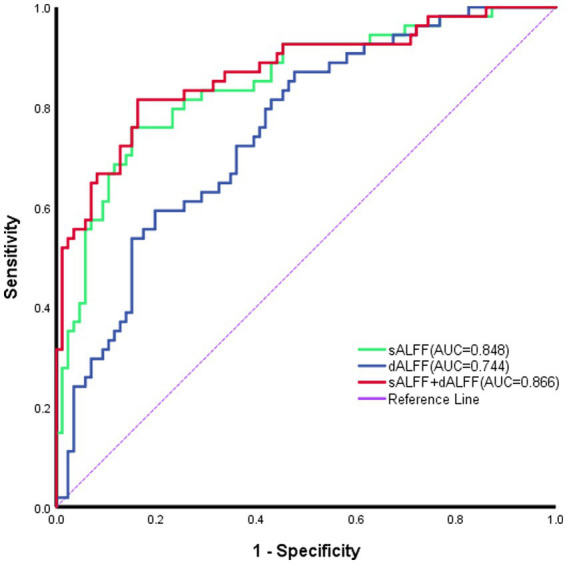
Receiver operating characteristic curves of sALFF or dALFF and their combination for identifying ASD. The areas under the curve (AUCs) for sALFF and dALFF values were 0.848 and0.744, respectively; and the AUC for their combination was 0.866. sALFF, static ALFF; dALFF, dynamic ALFF.

No notable correlation was found between sALFF or dALFF values in the left mOFC, left MTG, left PCu, left iOFC and the ABC/CARS scores in children with ASD (*r* = 0.095, *p* = 0.434; *r* = 0.000, *p* = 0.999; *r* = 0.063, *p* = 0.606; *r* = −0.006, *p* = 0.962; *r* = −0.040, *p* = 0.745; *r* = −0.128, *p* = 0.290; *r* = 0.057, *p* = 0.638; *r* = 0.080, *p* = 0.513; *r* = −0.001, *p* = 0.996; *r* = 0.113, *p* = 0.351) (Table 3 and Figures 3A–J). Then, ROC curve analysis was performed to evaluate the predictive efficiency of sALFF and dALFF. Results showed that the AUCs of sALFF and dALFF in abnormal brain regions for diagnosing ASD were 0.848 (*p* < 0.001, 95% CI: 0.781–0.916) and 0.744 (*p* < 0.001, 95% CI: 0.660–0.827), respectively. When sALFF and dALFF were combined, the AUC reached 0.866 (*p* < 0.001, 95% CI: 0.803–0.930) (Figure 4).

**Table 3:** Correlation analyses between ALFF and clinical scales.

		sALFF			dALFF	
		L mOFC	L MTG	L PCu	L iOFC	L MTG
	*r*	0.095	0.063	−0.040	0.057	−0.001
ABC	*p*	0.434	0.606	0.745	0.638	0.996
CARS	*r*	0.000	−0.006	−0.128	0.080	0.113
	*p*	0.999	0.962	0.290	0.513	0.351

L, left; sALFF, static amplitude of low frequency fluctuations; dALFF, dynamic amplitude of low frequency fluctuations; ABC, Autism Behavior Checklist; CARS, Childhood Autism Rating Scale, mOFC, medial orbitofrontal cortex; MTG, middle temporal gyrus; PCu, precuneus; iOFC, inferior orbitofrontal cortex.

## Discussion

Our study revealed that preschool children diagnosed with ASD exhibit reduced sALFF and/or dALFF in the left OFC, MTG, and PCu, in comparison to healthy controls. ROC analysis suggests a good discriminating ability of sALFF and dALFF in identifying ASD, where their combination showed a better efficiency. Although no correlation between sALFF or dALFF and their clinical scales was revealed, these results offer a deeper understanding of ASD-associated brain dysfunction in preschool children, which highlights the crucial role of MTG in the pathogenesis of ASD. Since most abnormal ALFF values are detected in the left hemisphere, the dominant side of included children, lateralization-related problems play an important role in the progression of ASD.

Our results showed that the left MTG in ASD group exhibited lower static and dynamic ALFF values. This supports the notion that MTG is crucial for brain functions in ASD patients. The MTG, which is responsible for “social brain” network, has often been associated with ASD across multiple studies (Amaral et al., 2008; Ogawa et al., 2019; Pelphrey and Carter, 2008; Sato et al., 2017). While MTG is closely associated with language, emotion, and social cognition, its alteration is significantly correlated with ASD (Xu J. et al., 2019). Similarly, reduced regional connectivity in right MTG is revealed in high-functioning adults with ASD (Itahashi et al., 2015). Although these studies emphasized the functional abnormalities in MTG, their conclusions significantly varied. For example, an increased ALFF value in right MTG is detected in low-functioning boys with ASD (Li G. et al., 2018), which is different from our findings. The discrepancy may be due to the fact that our study reported a difference in left MTG, which is distinct from right MTG as they reported. Moreover, the age, gender, sample sizes, IQ or ASD symptomatology, and investigation methods may result in different conclusions.

MTG is also an essential component of the semantic system located in the Wernicke region. It primarily facilitates semantic expression, serves as a storage site for lexical items (Hickok, 2012) and plays a key part in processing auditory information and comprehending language (Sugimoto et al., 2023). Specifically, the left MTG aids the left inferior frontal gyrus in comprehending semantics and accessing lexical details, participating in the selection of items according to their linguistic significance (Ogawa et al., 2019). Our research identified a decreased ALFF value in the left MTG in ASD group, aligning with earlier research indicating that ALFF is reduced in the left MTG and left inferior frontal gyrus in preschool-aged boys with ASD (Lan et al., 2021). This finding may indicate that the language functions of the MTG in individuals with ASD become more lateralized to the left hemisphere. Supporting this viewpoint, Xu J. et al. (2019) suggest that the left MTG is more actively involved in diverse cognitive activities linked to language processing and the acquisition of non-abstract semantic information compared to its right counterpart, thereby allowing the left MTG to convey semantic meanings more efficiently and thoroughly. Overall, these findings indicate that the reduced ALFF in the left MTG of preschoolers with ASD may be closely related to their core symptom, the impaired social interaction and language communication.

We also observed a reduction of sALFF in the left mOFC, as well as a decrease of dALFF in the left iOFC, suggesting a lowered level of intrinsic neural activity in these two areas in preschool patients with ASD. Both the mOFC and iOFC are part of the orbitofrontal cortex (OFC), which is a part of the prefrontal cortex (PFC)—an area notably involved in ASD—playing an essential role in social behaviors, including emotional regulation, task execution, social cognition and decision making (Liu et al., 2020; Rempel-Clower, 2007; Rudebeck and Rich, 2018). In particular, the mOFC is linked to complex emotions and the processing of rewards and punishments, both crucial for social interactions (Kandilarova et al., 2019). Prior study has validated that individuals with ASD display both anatomical (Zielinski et al., 2014) and functional (Green et al., 2015) changes in the OFC. Damage to this area can lead to symptoms associated with ASD, such as rigidity, obsessive-compulsive behaviors, anxiety, and social indifference (Girgis et al., 2007). An fMRI investigation involving adolescents with ASD indicated a link between diminished activation of the OFC and atypical reward-related decision making (Carlisi et al., 2017). Echoing our results, this implies that individuals with ASD might begin to modify their reward processing during childhood, which impacts their capability to assess values and leads to repetitive and stereotypic behaviors (McAlonan et al., 2002; Lan et al., 2021). In contrast to our results, Yue et al. (2023) noted a significant increased dALFF within the iOFC in adults with ASD. This discrepancy may be due to the different age of the target population, where dALFF is lower in autistic children compared to adults. Another investigation using the ABIDE database (Karavallil Achuthan et al., 2022) also found that ASD children displayed higher ALFF values across various regions, particularly in the right OFC. The ABIDE database includes samples from multiple centers and diverse scanning conditions, which may lead to discrepant findings. In summary, taking into account the time-dependent fluctuations of neuronal activity, our research suggests that the functional changes in the OFC among individuals with ASD deserve more thorough exploration.

This research further identified a decline in sALFF within the left PCu. PCu is essential for advanced cognitive abilities, such as episodic memory, social cognition, consciousness, theory of mind, and self-reflection (Broyd et al., 2009; Raichle, 2015; Zhang and Li, 2012). A variety of neuroimaging investigations have suggested both structural and functional irregularities in PCu of ASD patients (Cavanna and Trimble, 2006; Assaf et al., 2010; Schneider et al., 2013; Valk et al., 2015). Previous studies have drawn different conclusions when evaluating the role of ALFF in ASD in subjects of different ages. For example, ALFF values of the right PCu are decreased in young children with ASD (Wu et al., 2023), while the dALFF value in the left PCu of adult patients with ASD showed a significant increase and exhibited a positive correlation with social emotional scores as well as total scores from the Autism Diagnostic Observation Schedule (ADOS) (Yue et al., 2023). This difference might arise from factors such as age, developmental stage, cognitive skills, and social capabilities of the participants. On the other hand, the PCu serves as an essential hub within the default mode network (DMN) of the human brain. ALFF in DMN areas has been implicated as a crucial predictor of social functioning (Guo et al., 2017; Utevsky et al., 2014). Reduced spontaneous activity in crucial regions of DMN may affect functional connectivity, which could, in turn, influence social processing in individuals with ASD. Our research indicated that children diagnosed with ASD exhibited lower sALFF levels in the PCu in comparison to TDs. This observation aligns with earlier studies highlighting the importance of the PCu in social cognition and underscore the crucial role of DMN in ASD development (Supekar et al., 2013; Padmanabhan et al., 2017).

To sum up, this study comprehensively elucidates the neurophysiological mechanisms behind all core symptoms of ASD (social impairment, language impairment, and stereotyped behavior) through the abnormal spontaneous brain activity identified by using static and dynamic ALFF. Previous studies have utilized various analysis methods of rs-fMRI, either relying on a single indicator or solely from a static or dynamic perspective, to investigate the potential neurophysiological mechanisms underlying the core symptoms of ASD. Itahashi et al. (2015) employed a graph theory framework integrating fALFF analysis in high-functioning ASD populations, specifically highlighting that disrupted local connectivity in the MTG constitutes a key neural substrate underlying social impairments. Guo et al. (2017) discovered that the ALFF value in the right PCu significantly decreased across all age groups, suggesting that abnormal spontaneous brain activity may serve as a potential mechanism underlying social deficits. Lan et al. (2021) utilized the ReHo method and found that the ReHo values in language-related brain regions, including the left MTG, were reduced in preschool boys. However, the fALFF, ALFF or ReHo index was deemed singular and unreliable, emphasizing the mechanisms involved in social deficits or language development disorders. Two recent studies have applied the methods of dALFF and dReHo to investigate the neurophysiological mechanisms underlying ASD symptoms in both adults and toddlers, focusing on dynamic local brain activity. The former study reported a significant increase in dALFF variability in the left PCu and the right inferior frontal orbital region, with the temporal variability of dALFF in the left PCu positively correlating with scores of social communication ability, thereby underscoring the critical role of precuneus abnormalities in social deficits. The latter study found that the dALFF values in the right MTG and right PCu of the ASD group were significantly lower than those in the LDD group, indicating that the reduced ALFF values in these regions are associated with deficits in language processing.

Importantly, our study suggested that every brain region exhibiting abnormal ALFF values was located in the left hemisphere, indicating a potential predisposition toward left hemisphere dysfunction in preschoolers with ASD. A previous study using DTI that focused on children with ASD (Yin et al., 2021) identified a notable reduction in the integrity of white matter fiber tracts in the left hemisphere of preschoolers with ASD, which was thought to be reflected in unusual daily behaviors. This finding reinforces our conclusions from the standpoint of structural MRI. Abnormal lateralization of ALFF might reveal unusual brain development patterns in autistic children. The left and right brain hemispheres demonstrate functional specialization, where the left hemisphere is chiefly involved in language and logical reasoning, whereas the right hemisphere plays a greater role in spatial awareness and emotional processing. In children with autism, atypical lateralization may hinder the maturation of these functions, leading to related symptoms.

Regarding the differences in brain regions observed in the results obtained from the analysis using sALFF and dALFF methods in this study, it stems from the fact that they elucidate the abnormalities in spontaneous brain activity among ASD patients from distinct perspectives. Generally, sALFF reflected the cumulative effect of spontaneous brain activity over a certain period, whereas dALFF captured the instantaneous changes in spontaneous brain activity (Ma et al., 2020). Furthermore, we employed the mean values of sALFF and dALFF as features for identifying ASD by ROC analysis. AUC values for sALFF and dALFF were 0.848 and 0.744, respectively, and the AUC of their combination reached 0.866. As a result, the use of sALFF and dALFF could provide detailed and complementary imaging insights to elucidate localized spontaneous alterations in brain functionality among individuals with ASD.

In this research, we did not find a notable relationship between ALFF values and clinical assessments (ABC and CARS), which is in line with findings from earlier studies (Li G. et al., 2018; Wu et al., 2023). One possible rationale is that the preschool children with ASD participating in our research were quite young, so that the score of ABC (Krug et al., 1980) provided by parents and CARS (Schopler et al., 1980) evaluated by clinicians could be subjective. Exploring alternative methodologies or utilizing updated clinical scales might yield more informative data. The Autism Diagnostic Interview-Revised (ADI-R) and the Second Edition of the Autism Diagnostic Observation Schedule (ADOS-2) are the diagnostic criteria most frequently used for identifying ASD, with sensitivities recorded at 80% (95% CI, 79–82%) and 91% (95% CI, 90–92%), respectively (Hirota and King, 2023). Additional studies are needed to explore the correlation between ALFF and results from behavioral as well as neuropsychological assessments, and expand the array of psychometric variables incorporated in the research, to deepen our understanding of the clinical implications tied to alterations in ALFF.

Our investigation acknowledges a number of limitations. Firstly, there is the issue of sample size. As our study focuses on preschool children with ASD aged 2–6 years, the collected sample size is relatively limited. We plan to expand our case cohort in subsequent research and conduct gender-stratified analyses when warranted. Secondly, the elevated comorbidity rates associated with ASD, including depression, attention deficit hyperactivity disorder (ADHD), and anxiety, might limit the generalizability of the findings. Thirdly, the ways to determine the optimal duration for brain activity dynamics remain ambiguous, which continues to spark debate regarding the selection of a better window length. Finally, this investigation based solely on resting-state fMRI (rs-fMRI) data has inherent methodological constraints. In future studies, we aim to integrate task-based fMRI or multimodal neuroimaging methods when feasible. Additionally, we plan to leverage advanced machine learning and deep learning technologies to optimize brain parameter features derived from different analytical approaches. This comprehensive strategy will enhance our understanding of the underlying neural mechanisms of ASD and further improve the diagnostic accuracy of early ASD identification.

## Conclusion

In the current study, we employed sALFF and dALFF to investigate the aberrant spontaneous brain activity in children with ASD. The results revealed a decrease in ALFF values across various brain areas, including left MTG, left iOFC, left mOFC, and left PCu, which indicates a possible leftward shift of abnormal brain regions in preschoolers with ASD. These changes could potentially act as biological indicators for the early detection of ASD, emphasizing the crucial impact of impaired left MTG on ASD progression. Our findings suggest that analyzing static and dynamic ALFF may yield important insights into the neurophysiological mechanisms underlying ASD and ALFF may become a biomarker for identifying ASD at an early stage.

## Data Availability

The original contributions presented in the study are included in the article/Supplementary material, further inquiries can be directed to the corresponding authors.

## Footnotes

^1^ http://rfmri.org/dpabi

## References

[ref1] Amaral D. G. Schumann C. M. Nordahl C. W. (2008). Neuroanatomy of autism. Trends Neurosci. 31, 137–145. doi: 10.1016/j.tins.2007.12.005, PMID: 18258309

[ref2] American Psychiatric Association (2013). Diagnostic and statistical manual of mental disorders. 5th Edn. Washington, DC: American Psychiatric Association.

[ref3] Assaf M. Jagannathan K. Calhoun V. D. Miller L. Stevens M. C. Sahl R.. (2010). Abnormal functional connectivity of default mode sub-networks in autism spectrum disorder patients. NeuroImage 53, 247–256. doi: 10.1016/j.neuroimage.2010.05.067, PMID: 20621638 PMC3058935

[ref4] Broyd S. J. Demanuele C. Debener S. Helps S. K. James C. J. Sonuga-Barke E. J. (2009). Default-mode brain dysfunction in mental disorders: a systematic review. Neurosci. Biobehav. Rev. 33, 279–296. doi: 10.1016/j.neubiorev.2008.09.002, PMID: 18824195

[ref5] Carlisi C. O. Norman L. Murphy C. M. Christakou A. Chantiluke K. Giampietro V.. (2017). Comparison of neural substrates of temporal discounting between youth with autism spectrum disorder and with obsessive-compulsive disorder. Psychol. Med. 47, 2513–2527. doi: 10.1017/s0033291717001088, PMID: 28436342 PMC5964452

[ref6] Cavanna A. E. Trimble M. R. (2006). The precuneus: a review of its functional anatomy and behavioural correlates. Brain 129, 564–583. doi: 10.1093/brain/awl004, PMID: 16399806

[ref7] Cui Q. Sheng W. Chen Y. Pang Y. Lu F. Tang Q.. (2019). Dynamic changes of amplitude of low-frequency fluctuations in patients with generalized anxiety disorder. Hum. Brain Mapp. 41, 1667–1676. doi: 10.1002/hbm.24902, PMID: 31849148 PMC7267950

[ref8] Di Martino A. Yan C. G. Li Q. Denio E. Castellanos F. X. Alaerts K.. (2013). The autism brain imaging data exchange: towards a large-scale evaluation of the intrinsic brain architecture in autism. Mol. Psychiatry 19, 659–667. doi: 10.1038/mp.2013.78, PMID: 23774715 PMC4162310

[ref9] Ecker C. Bookheimer S. Y. Murphy D. G. M. (2015). Neuroimaging in autism spectrum disorder: brain structure and function across the lifespan. Lancet Neurol. 14, 1121–1134. doi: 10.1016/s1474-4422(15)00050-2, PMID: 25891007

[ref10] Fonov V. Evans A. C. Botteron K. Almli C. R. McKinstry R. C. Collins D. L. (2011). Unbiased average age-appropriate atlases for pediatric studies. NeuroImage 54, 313–327. doi: 10.1016/j.neuroimage.2010.07.033, PMID: 20656036 PMC2962759

[ref11] Fu Z. Tu Y. Di X. Du Y. Pearlson G. D. Turner J. A.. (2018). Characterizing dynamic amplitude of low-frequency fluctuation and its relationship with dynamic functional connectivity: an application to schizophrenia. NeuroImage 180, 619–631. doi: 10.1016/j.neuroimage.2017.09.035, PMID: 28939432 PMC5860934

[ref12] Girgis R. R. Minshew N. J. Melhem N. M. Nutche J. J. Keshavan M. S. Hardan A. Y. (2007). Volumetric alterations of the orbitofrontal cortex in autism. Prog. Neuro-Psychopharmacol. Biol. Psychiatry 31, 41–45. doi: 10.1016/j.pnpbp.2006.06.007, PMID: 16863674 PMC2888006

[ref13] Green S. A. Hernandez L. Tottenham N. Krasileva K. Bookheimer S. Y. Dapretto M. (2015). Neurobiology of sensory overresponsivity in youth with autism Spectrum disorders. JAMA Psychiatry 72, 778–786. doi: 10.1001/jamapsychiatry.2015.0737, PMID: 26061819 PMC4861140

[ref14] Guo X. Chen H. Long Z. Duan X. Zhang Y. Chen H. (2017). Atypical developmental trajectory of local spontaneous brain activity in autism spectrum disorder. Sci. Rep. 7:39822. doi: 10.1038/srep39822, PMID: 28057930 PMC5216408

[ref15] Hickok G. (2012). The cortical organization of speech processing: feedback control and predictive coding the context of a dual-stream model. J. Commun. Disord. 45, 393–402. doi: 10.1016/j.jcomdis.2012.06.004, PMID: 22766458 PMC3468690

[ref16] Hirota T. King B. H. (2023). Autism Spectrum disorder: a review. JAMA 329, 157–168. doi: 10.1001/jama.2022.23661 36625807

[ref17] Itahashi T. Yamada T. Watanabe H. Nakamura M. Ohta H. Kanai C.. (2015). Alterations of local spontaneous brain activity and connectivity in adults with high-functioning autism spectrum disorder. Mol. Autism. 6:30. doi: 10.1186/s13229-015-0026-z, PMID: 26023326 PMC4446946

[ref18] James M. S. Michael B. Peter T. B. Kaylena A. E. M. Richard S. Oluwasanmi K.. (2019). Human cognition involves the dynamic integration of neural activity and neuromodulatory systems. Nat. Neurosci. 22, 289–296. doi: 10.1038/s41593-018-0312-0, PMID: 30664771

[ref19] Jenkinson M. Bannister P. Brady M. Smith S. (2002). Improved optimization for the robust and accurate linear registration and motion correction of brain images. NeuroImage 17, 825–841. doi: 10.1016/s1053-8119(02)91132-8, PMID: 12377157

[ref20] Kandilarova S. Stoyanov D. Sirakov N. Maes M. Specht K. (2019). Reduced grey matter volume in frontal and temporal areas in depression: contributions from voxel-based morphometry study. Acta Neuropsychiatr. 31, 252–257. doi: 10.1017/neu.2019.20, PMID: 31234950

[ref21] Karavallil Achuthan S. Coburn K. L. Beckerson M. E. Kana R. K. (2022). Amplitude of low frequency fluctuations during resting state fMRI in autistic children. Autism Res. 16, 84–98. doi: 10.1002/aur.2846, PMID: 36349875

[ref22] Krug D. A. Arick J. Almond P. (1980). Behavior checklist for identifying severely handicapped individuals with high levels of autistic behavior. J. Child Psychol. Psychiatry 21, 221–229. doi: 10.1111/j.1469-7610.1980.tb01797.x, PMID: 7430288

[ref23] Küblböck M. Woletz M. Höflich A. Sladky R. Kranz G. S. Hoffmann A.. (2014). Stability of low-frequency fluctuation amplitudes in prolonged resting-state fMRI. NeuroImage 103, 249–257. doi: 10.1016/j.neuroimage.2014.09.038, PMID: 25251869

[ref24] Lan Z. Xu S. Wu Y. Xia L. Hua K. Li M.. (2021). Alterations of regional homogeneity in preschool boys with autism spectrum disorders. Front. Neurosci. 15:644543. doi: 10.3389/fnins.2021.644543, PMID: 33828452 PMC8019812

[ref26] Li J. Chen X. Zheng R. Chen A. Zhou Y. Ruan J. (2021). Altered cerebellum spontaneous activity in juvenile autism Spectrum disorders associated with clinical traits. J. Autism Dev. Disord. 52, 2497–2504. doi: 10.1007/s10803-021-05167-6, PMID: 34184142

[ref27] Li J. Duan X. Cui Q. Chen H. Liao W. (2018). More than just statics: temporal dynamics of intrinsic brain activity predicts the suicidal ideation in depressed patients. Psychol. Med. 49, 852–860. doi: 10.1017/s0033291718001502, PMID: 29909788

[ref28] Li G. Rossbach K. Jiang W. Du Y. (2018). Resting-state brain activity in Chinese boys with low functioning autism spectrum disorder. Ann. General Psychiatry 17:47. doi: 10.1186/s12991-018-0217-z, PMID: 30473720 PMC6234582

[ref29] Liang G. Li X. Yuan H. Sun M. Qin S. Wei B. (2023). Abnormal static and dynamic amplitude of low-frequency fluctuations in multiple brain regions of methamphetamine abstainers. Math. Biosci. Eng. 20, 13318–13333. doi: 10.3934/mbe.2023593, PMID: 37501489

[ref30] Liu X. Bautista J. Liu E. Zikopoulos B. (2020). Imbalance of laminar-specific excitatory and inhibitory circuits of the orbitofrontal cortex in autism. Mol. Autism. 11:83. doi: 10.1186/s13229-020-00390-x, PMID: 33081829 PMC7574354

[ref31] Ma M. Zhang H. Liu R. Liu H. Yang X. Yin X.. (2020). Static and dynamic changes of amplitude of low-frequency fluctuations in cervical discogenic pain. Front. Neurosci. 14:733. doi: 10.3389/fnins.2020.00733, PMID: 32760245 PMC7372087

[ref32] McAlonan G. M. Daly E. Kumari V. Critchley H. D. van Amelsvoort T. Suckling J.. (2002). Brain anatomy and sensorimotor gating in Asperger's syndrome. Brain 125, 1594–1606. doi: 10.1093/brain/awf150, PMID: 12077008

[ref33] Mei T. Ma Z.-H. Guo Y.-Q. Lu B. Cao Q.-J. Chen X.. (2022). Frequency-specific age-related changes in the amplitude of spontaneous fluctuations in autism. Transl. Pediatr. 11, 349–358. doi: 10.21037/tp-21-412, PMID: 35378963 PMC8976680

[ref34] Niu X. Gao X. Zhang M. Dang J. Sun J. Lang Y.. (2023). Static and dynamic changes of intrinsic brain local connectivity in internet gaming disorder. BMC Psychiatry 23:578. doi: 10.1186/s12888-023-05009-y, PMID: 37558974 PMC10410779

[ref35] Ogawa R. Kagitani-Shimono K. Matsuzaki J. Tanigawa J. Hanaie R. Yamamoto T.. (2019). Abnormal cortical activation during silent reading in adolescents with autism spectrum disorder. Brain and Development 41, 234–244. doi: 10.1016/j.braindev.2018.10.013, PMID: 30448302

[ref36] Padmanabhan A. Lynch C. J. Schaer M. Menon V. (2017). The default mode network in autism. Biol. Psychiatry Cogn. Neurosci. Neuroimaging 2, 476–486. doi: 10.1016/j.bpsc.2017.04.004, PMID: 29034353 PMC5635856

[ref37] Pelphrey K. A. Carter E. J. (2008). Charting the typical and atypical development of the social brain. Dev. Psychopathol. 20, 1081–1102. doi: 10.1017/s0954579408000515, PMID: 18838032

[ref38] Raichle M. E. (2015). The brain's default mode network. Annu. Rev. Neurosci. 38, 433–447. doi: 10.1146/annurev-neuro-071013-014030, PMID: 25938726

[ref39] Rempel-Clower N. L. (2007). Role of orbitofrontal cortex connections in emotion. Ann. N. Y. Acad. Sci. 1121, 72–86. doi: 10.1196/annals.1401.026, PMID: 17846152

[ref40] Rudebeck P. H. Rich E. L. (2018). Orbitofrontal cortex. Curr. Biol. 28, R1083–r1088. doi: 10.1016/j.cub.2018.07.018, PMID: 30253144 PMC9253859

[ref41] Sato W. Kochiyama T. Uono S. Yoshimura S. Kubota Y. Sawada R.. (2017). Reduced gray matter volume in the social brain network in adults with autism spectrum disorder. Front. Hum. Neurosci. 11:395. doi: 10.3389/fnhum.2017.00395, PMID: 28824399 PMC5543091

[ref42] Saxena R. Babadi M. Namvarhaghighi H. Roullet F. I. (2020). Role of environmental factors and epigenetics in autism spectrum disorders. Prog. Mol. Biol. Transl. Sci. 173, 35–60. doi: 10.1016/bs.pmbts.2020.05.002, PMID: 32711816

[ref43] Schneider K. Pauly K. D. Gossen A. Mevissen L. Michel T. M. Gur R. C.. (2013). Neural correlates of moral reasoning in autism spectrum disorder. Soc. Cogn. Affect. Neurosci. 8, 702–710. doi: 10.1093/scan/nss051, PMID: 22569187 PMC3739915

[ref44] Schopler E. Reichler R. J. RF D. V. Daly K. (1980). Toward objective classification of childhood autism: Childhood Autism Rating Scale (CARS). J. Autism Dev. Disord. 10, 91–103. doi: 10.1007/BF02408436 6927682

[ref45] Song J. Lei T. Li Y. Zhou L. Yan W. Li H.. (2023). Dynamic alterations in the amplitude of low-frequency fluctuation in patients with cerebral small vessel disease. Front. Mol. Neurosci. 16:1200756. doi: 10.3389/fnmol.2023.1200756, PMID: 37808469 PMC10556663

[ref46] Sugimoto H. Abe M. S. Otake-Matsuura M. (2023). Word-producing brain: contribution of the left anterior middle temporal gyrus to word production patterns in spoken language. Brain Lang. 238:105233. doi: 10.1016/j.bandl.2023.105233, PMID: 36842390

[ref47] Sun X. Allison C. Wei L. Matthews F. E. Auyeung B. Wu Y. Y.. (2019). Autism prevalence in China is comparable to Western prevalence. Mol. Autism. 10:7. doi: 10.1186/s13229-018-0246-0, PMID: 30858963 PMC6394100

[ref48] Supekar K. Uddin L. Q. Khouzam A. Phillips J. Gaillard W. D. Kenworthy L. E.. (2013). Brain hyperconnectivity in children with autism and its links to social deficits. Cell Rep. 5, 738–747. doi: 10.1016/j.celrep.2013.10.001, PMID: 24210821 PMC3894787

[ref49] Utevsky A. V. Smith D. V. Huettel S. A. (2014). Precuneus is a functional core of the default-mode network. J. Neurosci. 34, 932–940. doi: 10.1523/jneurosci.4227-13.2014, PMID: 24431451 PMC3891968

[ref50] Valk S. L. Di Martino A. Milham M. P. Bernhardt B. C. (2015). Multicenter mapping of structural network alterations in autism. Hum. Brain Mapp. 36, 2364–2373. doi: 10.1002/hbm.22776, PMID: 25727858 PMC6129398

[ref51] Wiśniowiecka-Kowalnik B. Nowakowska B. A. (2019). Genetics and epigenetics of autism spectrum disorder-current evidence in the field. J. Appl. Genet. 60, 37–47. doi: 10.1007/s13353-018-00480-w, PMID: 30627967 PMC6373410

[ref52] Wu S. Wen Z. Yang W. Jiang C. Zhou Y. Zhao Z.. (2023). Potential dynamic regional brain biomarkers for early discrimination of autism and language development delay in toddlers. Front. Neurosci. 16:1097244. doi: 10.3389/fnins.2022.1097244, PMID: 36699523 PMC9869111

[ref53] Xu S. Li M. Yang C. Fang X. Ye M. Wei L.. (2019). Altered functional connectivity in children with low-function autism spectrum disorders. Front. Neurosci. 13:806. doi: 10.3389/fnins.2019.00806, PMID: 31427923 PMC6688725

[ref54] Xu J. Wang C. Xu Z. Li T. Chen F. Chen K.. (2019). Specific functional connectivity patterns of middle temporal gyrus subregions in children and adults with autism spectrum disorder. Autism Res. 13, 410–422. doi: 10.1002/aur.2239, PMID: 31729198

[ref55] Yan C.-G. Wang X.-D. Zuo X.-N. Zang Y.-F. (2016). DPABI: data processing & analysis for (resting-state) brain imaging. Neuroinformatics 14, 339–351. doi: 10.1007/s12021-016-9299-4, PMID: 27075850

[ref56] Yang Y. Zhao R. Zhang F. Bai R. Li S. Cui R.. (2022). Dynamic changes of amplitude of low-frequency in systemic lupus erythematosus patients with cognitive impairment. Front. Neurosci. 16:929383. doi: 10.3389/fnins.2022.929383, PMID: 36081656 PMC9447953

[ref57] Yi T. Ji C. Wei W. Wu G. Jin K. Jiang G. (2024). Cortical-cerebellar circuits changes in preschool ASD children by multimodal MRI. Cereb. Cortex 34:bhae090. doi: 10.1093/cercor/bhae090, PMID: 38615243

[ref58] Yin Y. Xu S. Li C. Li M. Liu M. Yan J.. (2021). Association of reduced tract integrity with social communication deficits in preschool autism children: a tract-based spatial statistics study. Neuropsychiatr. Dis. Treat. 17, 2003–2010. doi: 10.2147/NDT.S306596 34168457 PMC8219119

[ref59] Yue X. Shen Y. Li Y. Zhang G. Li X. Wei W.. (2023). Regional dynamic neuroimaging changes of adults with autism spectrum disorder. Neuroscience 523, 132–139. doi: 10.1016/j.neuroscience.2023.04.016, PMID: 37270101

[ref60] Yue X. Zhang G. Li X. Shen Y. Wei W. Bai Y.. (2022). Brain functional alterations in Prepubertal boys with autism Spectrum disorders. Front. Hum. Neurosci. 16:891965. doi: 10.3389/fnhum.2022.891965, PMID: 35664346 PMC9160196

[ref61] Yu-Feng Z. Yong H. Chao-Zhe Z. Qing-Jiu C. Man-Qiu S. Meng L.. (2007). Altered baseline brain activity in children with ADHD revealed by resting-state functional MRI. Brain Dev 29, 83–91. doi: 10.1016/j.braindev.2006.07.002, PMID: 16919409

[ref62] Zhang S. Li C. S. (2012). Functional connectivity mapping of the human precuneus by resting state fMRI. NeuroImage 59, 3548–3562. doi: 10.1016/j.neuroimage.2011.11.023, PMID: 22116037 PMC3288461

[ref63] Zheng R. Chen Y. Jiang Y. Wen M. Zhou B. Li S.. (2021). Dynamic altered amplitude of low-frequency fluctuations in patients with major depressive disorder. Front. Psych. 12:683610. doi: 10.3389/fpsyt.2021.683610, PMID: 34349681 PMC8328277

[ref64] Zhu Q. Q. Tian S. Zhang L. Ding H. Y. Gao Y. X. Tang Y.. (2024). Altered dynamic amplitude of low-frequency fluctuation in individuals at high risk for Alzheimer's disease. Eur. J. Neurosci. 59, 2391–2402. doi: 10.1111/ejn.16267, PMID: 38314647

[ref65] Zielinski B. A. Prigge M. B. Nielsen J. A. Froehlich A. L. Abildskov T. J. Anderson J. S.. (2014). Longitudinal changes in cortical thickness in autism and typical development. Brain 137, 1799–1812. doi: 10.1093/brain/awu083, PMID: 24755274 PMC4032101

[ref66] Zuo X.-N. Xing X.-X. (2014). Test-retest reliabilities of resting-state FMRI measurements in human brain functional connectomics: a systems neuroscience perspective. Neurosci. Biobehav. Rev. 45, 100–118. doi: 10.1016/j.neubiorev.2014.05.009, PMID: 24875392

